# German Beck Scale for Suicide Ideation (BSS): psychometric properties from a representative population survey

**DOI:** 10.1186/s12888-017-1559-9

**Published:** 2017-12-04

**Authors:** Sören Kliem, Anna Lohmann, Thomas Mößle, Elmar Brähler

**Affiliations:** 1 Criminological Research Institute of Lower Saxony, Lützerodestraße 9, 30161 Hannover, Germany; 2 State Police College of Baden-Wuerttemberg, Sturmbühlstraße 250, 78054 Villingen-Schwenningen, Germany; 3 Department of Psychosomatic Medicine and Psychotherapy, University of Mainz, Untere Zahlbacher Str. 8, 55131 Mainz, Germany; 4 Department of Medical Psychology and Medical Sociology, University of Leipzig, Philipp-Rosenthal-Str. 55, 04103 Leipzig, Germany

**Keywords:** Suicide ideation, Beck Scale for Suicide Ideation (BSS), Validation, Population sample, Measurement invariance, Psychometric analysis

## Abstract

**Background:**

Suicidal ideation has been identified as one of the major predictors of attempted or actual suicide. Routinely screening individuals for endorsing suicidal thoughts could save lives and protect many from severe psychological consequences following the suicide of loved ones. The aim of this study was to validate the German version of the Beck Scale for Suicide Ideation (BSS) in a sample representative for the Federal Republic of Germany.

**Methods:**

All 2450 participants completed the first part of the Scale, the BSS-Screen. A risk group of *n* = 112 individuals (4.6%) with active or passive suicidal ideation was identified and subsequently completed the entire BSS.

**Results:**

Satisfactory internal reliability (α = .97 for the BSS-Screen; α = .94 for the entire BSS) and excellent model fit indices for the one-dimensional factorial structure of the BSS-Screen (CFI = .998; TLI = .995; RMSEA = .045 [95%-CI: .030-.061]) were confirmed. Measurement invariance analyses supported strict invariance across gender, age, and depression status. We found correlations with related self-report measures in expected directions comparable to previous studies, indicating satisfactory construct validity.

**Limitations:**

Our study involved cross sectional data, hence neither predictive validity nor retest-reliability were examined. As only the risk group of *n* = 112 individuals completed the entire measure, confirmatory factor analyses could not be conducted for the full BSS.

**Conclusion:**

The German translation of the BSS is a reliable and valid instrument for assessing suicidal ideation in the general population. Using it as a screening device in general and specialized medical care could substantially advance suicide prevention.

## Background

The World Health Organization [1] estimated that in 2012 approximately 800,000 people died of suicide worldwide. In Germany, around 10,000 suicides are recorded each year, which is 2.5 times the number of motor vehicle deaths within the same period of time [2]. Each of these suicides itself constitutes a tragedy. On top of that, they also strongly affect society: Numerous other people are affected by every suicide and often need psychosocial support [3]. An increased risk of committing suicide has been found in individuals who have lost their partner [4] or their child to suicide [5]. Moreover, in the United States, loss of productivity resulting from suicides and suicide attempts mounts up to 11.8 billion dollars each year [6], stressing that prevention and early detection of suicidal behavior is of utmost importance.

Several risk factors for suicidal behavior have been identified, for example low socio- economic status, experienced child abuse, and mental disorders (e.g. [7–9]). Protective factors like religious affiliation, social support, life satisfaction, and having children [10, 11] are related to lower suicide rates. One of the major predictors for committing or attempting suicide is the occurrence of suicidal ideation (e.g. [10–14]). Within the first year after the onset of suicidal thoughts, the risk for attempted suicide increases by approximately 170 times [9] as the transition from suicidal thoughts to behavior is often implemented during this period of time.

Thus, a routine assessment of suicidal thinking as part of general and specialized medical care could substantially advance suicide prevention. Especially because many practitioners are treating suicidal individuals without realizing it: Half of the individuals committing suicide contact primary or specialized health care facilities within 4 weeks prior to their death [15]. However, extensive exploration of suicidality with every patient cannot be provided by primary health care as time and financial resources are limited. Instead of clinical interviews – as for example the Scale for Suicide Ideation (SSI) [16]) – less time-consuming inventories seem more practical.

The Beck Scale for Suicide Ideation (BSS) [17] is the self-report version of the interviewer-administered SSI [16] and is one of the most widely used self-report instruments for the assessment of suicidal thinking. It helps to identify suicidal individuals provided that they are willing to acknowledge and share their thoughts. The BSS serves as a routine screening for existent suicidal thinking (BSS-Screen) and can also aid in a more extensive exploration of the severity of such thoughts (total BSS score). It can be administered in various settings (e.g., psychiatric-psychotherapeutic care, general medical services, and forensic psychiatry) and the routine screening, consisting only of five items, can be regarded as very time-efficient.

The BSS has proven to be a reliable measure across many different settings and samples, showing good internal consistencies e.g. α = .87 in an outpatient sample [18], α = .89 in a risk sample [19], and α = .88 in a non-clinical student sample [20]. One-week retest reliabilities of *r*_*tt*_ = .54 [17] and *r*_*tt*_ = .88 [21] have been found. Suicidal ideation as measured by the BSS has been shown to be strongly associated with hopelessness (e.g. [22–24]) and depression (e.g. [22, 25, 26]). High correlations between the BSS and other instruments for the measurement of suicidality have also been found, for example with the Suicide Probability Scale [25], the Adult Suicidal Ideation Questionnaire [25], and the Ratings of Suicidal Thoughts [24], providing support for convergent validity.

Its convincing overall quality has made the BSS one of the major scales for the assessment of suicidal ideation worldwide. The measure exists in various translations including Korean (e.g. [27]), Chinese (e.g. [28]), French (e.g. [29]), Persian (e.g. [30]), Dutch (e.g. [31]), Malay (e.g. [32]), Norwegian [33], and Urdu [34].

Although it has been translated into several languages, so far, there has been no official German version. Thus, the aim of this study is to investigate the reliability, validity, factorial structure, and factorial invariance of the German BSS in a large German population sample.

## Methods

### Study design and participants

Commissioned by the University of Leipzig, an independent institute for opinion and social research (USUMA, Berlin) collected the data in 2014. Sampling was conducted using a threefold random selection procedure in the entire inhabited territory of the Federal Republic of Germany. Firstly, 258 non-overlapping regional areas in Germany were defined by use of Cox-allocations (an algorithm providing a random rounding procedure thus allowing for unbiased stratification). Secondly, target households were randomly selected within these areas through random route procedures and thirdly, the specific target person in the respective households was randomly determined among all household members aged 14 years or older, who were able to sufficiently understand written German language. The selection of the individual to be interviewed was carried out with the help of Kish selection, which is a pre-assigned table of random numbers that helps the interviewer to determine the household member to interview [35]. Written consent was provided by all participants. Each target person was individually interviewed at home by a trained interviewer and was asked to complete several self-report questionnaires. In accordance with the American manual, the BSS, however, was exclusively completed by participants aged at least 18 years. Proper conduct of the interviews was assessed by sending prestamped postcards to 38.7% of the participants. Approximately 53% of these postcards were returned, all of them affirmative.

Altogether 2527 individuals were interviewed by 206 interviewers which constitutes a response rate of 54.8%. Two thousand four hundred fifty individuals were aged 18 years and older, thus completed the BSS and were included in the following psychometric analysis. The participant’s mean age was *M* = 50.51 years (*SD* = 17.0) with a range of 18–95 years; *n* = 88 (3.6%) had nationalities other than German and 53.9% of the participants were female. Further sample details can be found in Table 1. The BSS-Screen identified *n* = 112 individuals (4.6%) with active or passive suicidal ideation, whose mean age was *M* = 49.7 years (*SD* = 17.83). 53% of them were male. In the following, they will be referred to as the “risk group”. All procedures were authorized by the Ethics Committee of the Medical Faculty of the University of Leipzig (Az.: 063-14-10,032,014).

**Table 1:** Demographic characteristics of the study sample

Sample characteristics	Men (*N* = 1130)	Women (*N* = 1320)	Total sample (*N* = 2450)
Age group, *N* (%)			
18-24	88 (7.8%)	93 (7.0%)	181 (7.4%)
25-34	150 (13.3%)	188 (14.2%)	338 (13.8%)
35-44	183 (16.2%)	218 (16.5%)	401 (16.4%)
45-54	221 (19.6%)	264 (20.0%)	485 (19.8%)
55-64	225 (19.9%)	251 (19.0%)	476 (19.4%)
65-74	177 (15.7%)	183 (13.9%)	360 (14.7%)
> 74	86 (7.6%)	123 (9.3%)	209 (8.5%)
Living with a partner, *N* (%)	702 (63.1%)	740 (57.2%)	1442 (59.9%)
Having at least 1 child, *N* (%)	208 (18.4%)	341 (25.8%)	549 (22.4%)
Member of a church, *N* (%)	770 (68.5%)	984 (74.8%)	1754 (71.9%)
Level of education attained, *N* (%)			
Completed Year 9	429 (38.0%)	454 (34.4%)	883 (36.0%)
Completed Year 10	412 (36.5%)	573 (43.5%)	985 (40.2%)
Completed Year 12	118 (10.4%)	120 (9.1%)	238 (9.7%)
University Degree	133 (11.8%)	122 (9.2%)	255 (10.4%)
Other	38 (3.4%)	51 (3.9%)	89 (3.5%)
Employment status, *N* (%)			
In Training	48 (4.3%)	47 (3.5%)	95 (3.8%)
Working (> 35 h)	605 (53.8%)	388 (29.5%)	993 (40.7%)
Working (< 35 h)	51 (4.5%)	316 (24.0%)	367 (15.0%)
Unemployed	74 (6.6%)	77 (5.9%)	151 (6.2%)
Homemaker	10 (0.9%)	93 (7.1%)	103 (4.2%)
Retired	334 (29.7%)	377 (28.6%)	711 (29.1%)
Other	2 (0.2%)	18 (1.4%)	20 (0.8%)
Missing	6 (0.5%)	4 (0.3%)	10 (0.4%)
Monthly household income in €, *N* (%)			
< 1250	168 (14.9%)	291 (22.0%)	459 (18.7%)
1250 - 2000	299 (26.5%)	365 (27.7%)	664 (27.1%)
> 2000	633 (56.0%)	630 (47.7%)	1263 (51.6%)
Missing	30 (2.7%)	34 (2.6%)	64 (2.6%)

### Measures

#### Beck Scale for Suicide Ideation (BSS)

The BSS contains 21 statement groups each assessing various aspects of suicidal ideation (see Table 3). Each statement group consists of three sentences that describe different intensities of suicidal ideation, representing a three-point scale (0 to 2). Participants are instructed to choose the particular statement of each group that is most applicable to them. The total BSS score can range from 0 to 38, with higher values indicating a greater risk of suicide. Beck and Steer [17] do not distinguish different degrees of suicidal risk. Nor do they report a cutoff criterion as even very low total scores can be associated with elevated risks of suicide [36]. The first five items of the BSS serve as a screening device for suicidal ideation during the last week (including the day of assessment) and are summed up to the BSS-Screen score. Two filter questions (the statement groups four and five) assess the presence of active or passive suicidal thoughts. If participants endorse one of them (i.e., chose a sentence rated 1 or 2), they are to complete the subsequent 14 statement groups which allow for an assessment of the severity of existing suicidal ideation. If participants choose the response option rated “0” for both item 4 and item 5 they skip items 6 to 19 and precede to the last two statement groups. These last two items address frequency and intensity of former suicide attempts and are again to be answered by all participants. They are not part of the total BSS score. The translation of the German version (Beck-Suizidgedanken-Skala, BSS) was based on the WHO guidelines on translation and adaptation of psychometric instruments [37].

#### Patient Health Questionnaire 2 (PHQ-2)

The PHQ-2 [38] is a two-item self-administered depression module, which includes the two main criteria for major depression from the Diagnostic and Statistical Manual of Mental Disorders (5th ed., DSM-5) [39] rated on a scale from 0 = *not at all* to 3 = *nearly every day*. PHQ-2 sum scores range from 0 to 6, with higher values indicating more depressive symptomatology. A total score of ≥ 3 proved to be most suitable regarding sensitivity and specificity for the tentative diagnosis of major depressive disorder (sensitivity: 87%, specificity: 78%) and any other depressive disorder (sensitivity: 79%, specificity: 86%) [40]. The PHQ-2 showed high internal consistency in a recent population-based study (α = .75) [41].

#### Life satisfaction questionnaire (FLZ-8)

In order to assess life satisfaction, we used the FLZ-8, a shortened version of the *Fragebogen zur Lebenszufriedenheit* (FLZ) [Life Satisfaction Questionnaire] by Fahrenberg et al. [42]. It is used for the assessment of individual satisfaction in eight areas of life (friends/acquaintances, leisure time/hobbies, health, income/financial security, job/work, living situation, family life/children, relationship/sexuality) which can be summed up to an index of global life satisfaction. Participants are asked to rate their satisfaction in each area on a five-point scale ranging from 1 = *dissatisfied* to 5 = *very satisfied*, with higher values indicating higher life satisfaction. The FLZ showed good internal consistency in similar population-based surveys (α = .82) [42].

#### Beck Hopelessness Scale (BHS)

The BHS [43] is a 20-item scale measuring negative attitudes about the future. For each of nine optimistic and 11 pessimistic statements participants are asked to report whether it describes their attitude during the last week (*true*) or not (*false*). Scores range between 0 and 20, with higher values indicating greater hopelessness. In the present study, the BHS showed high internal consistency, α = .87.

### Statistical analyses

Internal consistency of the BSS is reported as coefficient α. Item-total correlations were determined correlating the respective item with the sum of all other items. Item difficulty (*P*_*i*_) coefficients were calculated as quotients of the sum of the item values that were obtained and the sum of the maximum achievable item values, multiplied by 100. To examine construct validity of the BSS, correlations with the PHQ-2, the FLZ-8, the BHS, and with the last two items of the BSS were calculated. We applied chained equation modeling [44] using the following variables: gender, age, monthly net income, educational status, and partnership status to estimate missing data (proportion of missing values of analyzed items: 0.1 – 0.4%) (see, for example, [45]). To avoid implausible item values, the estimated values ($\widehat{y}$) were corrected by predictive mean matching (i.e., the observable values closest to the predicted value were chosen). We used the R package mice [46] for imputation.

In order to verify whether the 5 items of the BSS-Screen may be summed up to one overall score (the BSS-Screen score), a one-factor model was tested using confirmatory factor analysis (CFA). Because of the three-point response format, maximum likelihood estimation was not considered appropriate [47]. Instead, we calculated a polychoric correlation matrix and used the mean- and variance-adjusted weighted least square estimator (WLMSV) [48] which has been found to be robust to violations of normality (e.g. [49]). Subsequently, goodness of fit was evaluated considering three different criteria and their respective cutoff values for a good model fit: the Comparative Fit Index (CFI > .950), Tucker Lewis Index (TLI > .950), and root mean square of approximation (RMSEA < .080). Because of the small size of the risk group within our non-clinical sample, no factor analysis concerning the entire BSS scale was performed.

Furthermore, we conducted several measurement invariance tests using multi-group factor analyses across gender (group 1: men; group 2: women), age (group 1: 18-34 years; group 2: 35-64 years; group 3: ≥ 65 years), depression status (group 1: < 3 sum score in PHQ-2; group 2: ≥ 3 sum score in PHQ-2). The groups were of the following sizes: gender: female *n* = 1230, male *n* = 1130; age: 18-34 years *n* = 519, 35-64 years *n* = 1362, ≥ 65 years *n* = 569; depression status: non-depressed *n* = 2227, possibly depressed *n* = 223. The same estimator as in the CFA (WLSMV) was used. These measurement invariance tests were performed using the sequential strategy discussed by Millsap and Yun-Tein [50]. As recommended by Chen [51], CFI differences with a cutoff value of Δ_CFI_ > .01 were used to test the different stages of measurement invariance. Data analysis was carried out with the R package lavaan [52].

## Results

### Item characteristics

Table 2 displays means, standard deviations, item difficulties, the frequency of item endorsement, and the corrected item-total correlation values for the five items of the BSS-Screen in the general population and in the risk group. Furthermore, the item characteristics of the entire BSS completed only by the risk group are listed in Table 3. The item-total correlation values in the general population, which ranged from *r*_*it*_ = .70 (active suicide attempt) to *r*_*it*_ = .78 (wish to live), can be regarded as very satisfactory. In the risk group, item-total correlation values were mostly satisfying (except for items 11 and 19).

**Table 2:** Item properties for the BSS-Screen and total scores

	General Population (*N* = 2450)					Risk group (*N* = 112)				
Item	*M*	*SD*	*P*_*i*_	%^a^	*r*_*it*_	*M*	*SD*	*P*_*i*_	%^a^	*r*_*it*_
1 Wish to live	0.05	0.24	3	3.8	.78	0.68	0.71	33	53.6	0.72
2 Wish to die	0.05	0.25	3	4.2	.78	0.67	0.69	33	54.5	0.80
3 Reasons for living or dying	0.045	0.24	3	3.8	.73	0.59	0.67	29	49.1	0.76
4 Active suicide attempt	0.02	0.18	1	2.0	.70	0.53	0.64	26	44.7	0.54
5 Passive suicide attempt	0.05	0.27	3	4.3	.71	1.18	0.51	58	94.6	0.44
BSS-Screen total score	0.22	0.98	2	–	–	3.64	2.54	36	–	–
Number of endorsed items^a^	0.18	0.75	–	–	–	2.96	1.58	–	–	–

*Note*. ^a^Item was regarded as endorsed if the statement rated 1 or 2 was chosen; *P*_i_ = item difficulty; *r*_*it*_ = item-rest correlation

**Table 3:** Item properties for the entire BSS and total scores in the risk group

Item	*M*	*SD*	*P*_*i*_	%	*r*_*it*_
1 Wish to live	0.68	0.71	34	53.6	.59
2 Wish to die	0.67	0.69	33	54.5	.61
3 Reasons for living or dying	0.59	0.67	29	49.1	.61
4 Active suicide attempt	0.53	0.64	26	44.6	.61
5 Passive suicide attempt	1.18	0.51	59	94.6	.37
6 Duration of suicidal thoughts	0.26	0.55	13	20.5	.55
7 Frequency of ideation	0.21	0.45	11	18.8	.62
8 Attitude toward ideation	0.67	0.66	33	56.3	.59
9 Control over suicidal action	0.23	0.46	11	21.4	.66
10 Deterrents to attempt	0.73	0.83	37	49.1	.47
11 Reasons for attempt	1.28	0.80	64	78.6	.19
12 Specificity of planning	0.30	0.58	15	24.1	.44
13 Availability or opportunity	0.37	0.67	18	25.9	.49
14 Capability to carry out attempt	0.54	0.63	27	46.4	.61
15 Expectancy of actual attempt	0.28	0.51	14	25.0	.72
16 Extent of actual preparation	0.15	0.45	7	11.6	.63
17 Suicide note	0.16	0.44	8	14.3	.58
18 Final acts	0.16	0.44	8	13.4	.43
19 Deception and concealment	0.84	0.75	42	62.5	.20
Total score	3.64	2.54	36	–	–
Number of endorsed items^a^	2.96	1.58	–	–	–

*Note*. ^a^Item was regarded as endorsed if the statement rated 1 or 2 was chosen. *P*_i_ = item difficulty; *r*_*it*_ = item-rest correlation

In the general population, *n* = 49 individuals (2.0%) reported one and *n* = 8 individuals (0.3%) reported several attempted suicides in their life. Their actual death wish during the suicide attempt was estimated as low by *n* = 20 individuals (35.1%), as moderate by *n* = 19 individuals (33.3%), and as strong by *n* = 18 individuals (31.6%).

### Internal consistency

Internal consistency based on the polychoric covariance matrix was computed as coefficient alpha. Considering that coefficient alpha could be affected by problems stemming from its assumptions not being met [53], we additionally computed McDonald’s omega. BSS-Screen for the general population yielded α = .97, ω = .97 and the entire BSS (for the risk group) showed an internal consistency of α = .94; ω = .94.

### Factorial validity

CFA revealed very good fit parameters for the one-factor model of the BSS-Screen. All assessed indices showed very good model fit for the total sample (CFI = .998; TLI = .995; RMSEA = .045 [95%-CI: .030-.061]). Thus, calculating a BSS-Screen score can be regarded as appropriate.

### Factorial invariance

The fit measures obtained in the measurement invariance analysis are presented in Table 4. Robust fit statistics are reported. Regarding the CFI differences, strict invariance can be assumed for gender, age and depression status, as for no group the cutoff value by Chen [51] was exceeded in any step.

**Table 4:** Fit indices for the measurement invariance

Model	χ^2^	*df*	CFI	RMSEA [95% CI]	Δ_CFI_	ΔRMSEA
Group = gender						
Configural	40.915	11	0.997	0.047 [0.032, 0.063]	–	–
Weak	48.435	15	0.997	0.043 [0.030, 0.056]	< .001	0.004
Strong	48.776	19	0.997	0.036 [0.024, 0.048]	< .001	0.007
Strict	50.061	23	0.998	0.031 [0.019, 0.043]	0.001	0.005
Group = age						
Configural	26.326	17	0.999	0.026 [0.000, 0.044]	–	–
Weak	57.155	25	0.997	0.040 [0.026, 0.053]	0.002	0.014
Strong	59.228	33	0.998	0.031 [0.018, 0.044]	0.001	0.009
Strict	61.958	41	0.998	0.025 [0.010, 0.037]	< .001	0.006
Group = depression status						
Configural	42.116	11	0.995	0.048 [0.033,0.064]	–	–
Weak	44.812	15	0.996	0.040 [0.027, 0.054]	0.001	0.008
Strong	53.68	19	0.995	0.039 [0.027, 0.051]	0.001	0.001
Strict	62.533	23	0.994	0.037 [0.026, 0.049]	0.001	0.002

*Note*. *CFI* Comparative Fit Index; All fit statistics are robust.; configural = (for identification purposes) loadings of one marker variable per factor fixed to 1, unique variances of marker variables fixed to 1; all thresholds equally constrained across groups, unique variance of first group fixed to 1, factor means of first group fixed to 0; weak/strong = additionally all free loadings constrained to equality across groups; strict = additionally all unique variances of all groups fixed to 1

### Construct validity

Correlation coefficients with related self-report instruments were calculated in order to determine evidence for validity of the German BSS. As can be seen in Table 5, there were substantial correlations between the BSS-Screen score and validity measures for the general population and the risk group in the expected directions: More severe suicidal thoughts were associated with higher depression (PHQ-2), lower life satisfaction (FLZ-8), higher degrees of hopelessness (BHS), higher numbers of suicide attempts and higher seriousness of the intent to die in these attempts. In the risk group, distinctively high associations between the total BSS score and the last two items of *r* = .53 were found.

**Table 5:** Correlation coefficients between the BSS-Screen, the total BSS score and other self-rating questionnaires

Measure	PHQ-2	FLZ-8	BHS	Suicide attempt	Death wish	BSS-Screen
Total sample						
BSS-Screen score	.33^***^	−.27^***^	.36^***^	.35^***^	.35^***^	–
Risk group						
BSS-Screen score	.26^**^	−.25^**^	.23^*^	.27^**^	.32^**^	–
Total BSS score	.31^**^	−.29^**^	.25^**^	.53^***^	.53^***^	.80^***^

*Note. PHQ-2* Patient Health Questionnaire 2, *FLZ-8* shortened version of the Life Satisfaction Questionnaire, *BHS* Beck Hopelessness Scale, BSS-Screen denotes the BSS-Screen score. Pearson’s correlation coefficient was used; ^*^ *p* < .05 (2-tailed), ^**^ *p* < .01 (2-tailed), ^***^ *p* < .001 (2-tailed)

## Discussion

In this study, we investigated the psychometric quality of the German BSS using a German representative population sample. The reliability of the measure was found to be satisfactory, meeting the requirement of α ≥ .80 for clinical measures. Our results are in line with coefficients α reported in former studies ranging from α = .75 to .98 (e.g. [21, 34, 54]). CFA confirmed the unidimensionality of the five BSS-Screen items, allowing a meaningful calculation of the BSS-Screen sum score. Measurement invariance analyses supported strict invariance across gender, age, and depression status, which allows for unbiased comparison of means, correlation coefficients, and path coefficients within structural equation models between the analyzed groups. As construct validity indices, all correlation coefficients between the BSS and related self-report instruments were in the expected directions and of substantial size. The association between the BSS and the BHS lies within the range of previous studies using non-clinical samples (*r* = .21 - .59) (e.g. [23, 33, 34]). Krajniak et al. [55] found a relation between the BSS and the PHQ-9 (the longer version of the PHQ-2) similar to ours (*r* = .37).

In our sample, suicidal thoughts were detected in 4.6% (95% CI[3,8%,5,5%]) of the general population. This number is below the prevalence rate of suicidal ideation found in previous studies: 8.0% of participants in a representative sample of the German general population reported suicidal ideation during the last 2 weeks [56], the WMH Survey [9] showed a lifetime prevalence of 9.7% for suicidal ideation in Germany and the European Study on the Epidemiology of Mental Disorders (ESEMED) [57] found lifetime prevalence of suicidal ideation of 9.8% in Germany. This could be explained by people higher on suicidality might having shown a lower return rate and therefore being under-represented in the final pool of participants. However, also in the three studies mentioned above, representative samples of adults were interviewed at home, filling out short questionnaires about their suicidality. The number of suicidal individuals consenting to participate should therefore not differ between these studies and ours. It might be more likely that the German BSS is a more conservative measure than the questionnaires employed in the aforementioned studies.

Unlike the rest of the BSS items, the statement groups 11 (*Reason for attempt*) and 19 (*Deception and concealment)* showed low item-total correlations (*r*_*it*_ = .19 and .20 respectively). This also occurred in the studies conducted by Beck and Steer [17] for the validation of the original BSS scale. As a clinical instrument, the BSS aims at assessing all relevant aspects of suicidal ideation and, considering the sensitive nature of the gathered information, its clinical utility should be top priority – even if this may be at the expense of psychometric quality. Therefore, these two items should always be included when assessing individuals who endorse the existence of suicidal ideation, regardless of their item characteristics. The reasons and behavior of individuals identified as being at risk of suicide can be of high interest to a therapist. When calculating sum scores, however, one might consider excluding these two items.

### Limitations

Despite the many strengths of this study, such as its large sample size and representativeness, certain limitations should be taken into consideration: First, the response rate was only 54.8%. However, in general population studies lower response rates than in clinical studies are quite common and the response rate of this study was comparable to similar general population surveys (e.g. [58–60]). Second, the diagnostic efficiency of the German BSS could not be studied because no additional clinical interviews were conducted whose results could have been compared to those of the BSS. Yet, previous studies have already found high correlations between the BSS and clinical interviews as the SSI (e.g., *r* = .90) [54]. Third, as we exclusively relied on self-report, it would be interesting for future studies to include behavioral or archival data as well. Forth, given the fact that only *n* = 112 individuals endorsed the screening items and therefore completed the entire BSS, CFA could not be conducted for the complete scale. In future research, investigators should therefore use the German BSS in larger clinical samples more prone to suicidal ideation as for instance depressive patients. Fifth, while this study incorporated several measures for the assessment of convergent validity, divergent validity was not sufficiently covered. Sixth, as this study solely involved cross sectional data, it neither addressed predictive validity nor test-retest reliability. These should be examined by future research as well.

## Conclusion

In summary, the German BSS has proved to be a reliable and valid screening instrument that could certainly help to identify more suicidal individuals in primary or specialized health care facilities. Our study in a very large German population sample showed that the BSS can be administered in this country, contributing evidence that it is an instrument appropriate for many different cultures. An interesting question for future research could be the assessment of cross-cultural invariance of the BSS.
